# Amino Acids and mTOR Mediate Distinct Metabolic Checkpoints in Mammalian G1 Cell Cycle

**DOI:** 10.1371/journal.pone.0074157

**Published:** 2013-08-19

**Authors:** Mahesh Saqcena, Deepak Menon, Deven Patel, Suman Mukhopadhyay, Victor Chow, David A. Foster

**Affiliations:** Department of Biological Sciences, Hunter College of the City University of New York, New York, New York, United States of America; Institut de Génétique et Développement de Rennes, France

## Abstract

**Objective:**

In multicellular organisms, cell division is regulated by growth factors (GFs). In the absence of GFs, cells exit the cell cycle at a site in G1 referred to as the restriction point (R) and enter a state of quiescence known as G0. Additionally, nutrient availability impacts on G1 cell cycle progression. While there is a vast literature on G1 cell cycle progression, confusion remains – especially with regard to the temporal location of R relative to nutrient-mediated checkpoints. In this report, we have investigated the relationship between R and a series of metabolic cell cycle checkpoints that regulate passage into S-phase.

**Methods:**

We used double-block experiments to order G1 checkpoints that monitor the presence of GFs, essential amino acids (EEAs), the conditionally essential amino acid glutamine, and inhibition of mTOR. Cell cycle progression was monitored by uptake of [^3^H]-thymidine and flow cytometry, and analysis of cell cycle regulatory proteins was by Western-blot.

**Results:**

We report here that the GF-mediated R can be temporally distinguished from a series of late G1 metabolic checkpoints mediated by EAAs, glutamine, and mTOR – the mammalian/mechanistic target of rapamycin. R is clearly upstream from an EAA checkpoint, which is upstream from a glutamine checkpoint. mTOR is downstream from both the amino acid checkpoints, close to S-phase. Significantly, in addition to GF autonomy, we find human cancer cells also have dysregulated metabolic checkpoints.

**Conclusion:**

The data provided here are consistent with a GF-dependent mid-G1 R where cells determine whether it is appropriate to divide, followed by a series of late-G1 metabolic checkpoints mediated by amino acids and mTOR where cells determine whether they have sufficient nutrients to accomplish the task. Since mTOR inhibition arrests cells the latest in G1, it is likely the final arbiter for nutrient sufficiency prior to committing to replicating the genome.

## Introduction

The vast majority of mutations that contribute to cancer cell proliferation and survival are in genes that regulate progression through G1 phase of the cell cycle [1,2]. A key regulatory site in G1 is the growth factor (GF)-dependent restriction point (R), originally described by Pardee [3], where cells receive permissive signals to progress through G1 and divide. In the absence of GFs, cells enter a quiescent state known as G0. This GF-dependent R has been mapped to a site about 3 to 4hr post-mitosis in virtually all mammalian cells examined [4]. In addition to GF signals, nutrient availability and mTOR (mammalian/mechanistic target of rapamycin) also impact on G1 cell cycle progression [5,6]. Several texts have suggested that R in mammalian cells is analogous to START in the yeast cell cycle. However, yeasts are single cell organisms that divide in response to nutrient availability, not GFs. Moreover TOR-regulated START responds to nutrient availability [7–9]. We have hypothesized a distinct “Cell Growth” checkpoint in late G1, where cells ensure the availability of adequate raw materials before committing to replicating the genome and dividing [2]. Thus, START is evolutionarily more related to the proposed Cell Growth checkpoint rather than the GF-mediated R.

In this report, we demonstrate that R and nutritional checkpoints mediated by essential amino acids (EAA), glutamine (Q), and mTOR are distinct and temporally distinguishable. We also demonstrate that in addition to GF autonomy, nutrient sensing in G1 is dysregulated in cancer cells resulting in S- and G2/M-phase arrest. In addition to revealing differences between R and nutrient-sensitive checkpoints, our data suggest that metabolic dysregulation provides novel opportunities for therapeutic intervention.

## Materials and Methods

### Materials

Reagents were obtained from the following sources: Antibodies against Akt, phospho-Akt (T308 and S473), S6K, phospho-S6K (T389), 4EBP1, phospho-4EBP1 (T37/46), LC3-II, Rb, phospho-Rb (T807/811), cyclin E, and actin were obtained from Cell Signaling; antibody against p21 was obtained from Santa Cruz Biotechnology; antibody against cyclin D was obtained from BD Biosciences; and anti-mouse and anti-rabbit HRP conjugated secondary antibodies were obtained from Promega. DMEM (D6429), DMEM lacking Gln (D5546), DMEM lacking Arg, Leu and Lys (D9443), dialyzed fetal bovine serum (DFBS) (F0392), and glutamine (G7513) were obtained from Sigma. Rapamycin was obtained from LC Laboratories, and Torin1 was obtained from Tocris. Ultima Gold scintillation fluid (6013681) and [^3^H]-thymidine deoxyribose (TdR) (20 Ci/mMol, 1 mCi/ml) (NET-027E) were obtained from PerkinElmer.

### Cells and cell culture conditions

BJ hTERT, MCF7, MDA-MB-231, and Panc-1 cells were obtained from the American Tissue Type Culture Collection. All the cells were maintained in Dulbecco’s modified Eagle medium (DMEM) supplemented with 10% fetal bovine serum (FBS) (Sigma).

### Western blot analysis

Proteins were extracted from cultured cells in M-PER (Thermo Scientific, 78501). Equal amounts of proteins were subjected to SDS-PAGE on polyacrylamide separating gels. Electrophoresed proteins were then transferred to nitrocellulose membrane. After transfer, membranes were blocked in an isotonic solution containing 5% non-fat dry milk in PBS. Membranes were then incubated with primary antibodies as described in the text. Depending on the origin of the primary antibody, either anti-mouse or anti-rabbit HRP conjugated IgG was used for detection using ECL system (Pierce).

### Thymidine incorporation assay

To determine the progression from G1 to S-phase, cells were labeled with 1µCi/ml [^3^H]-thymidine (TdR). At indicated times, cells were washed twice with 1ml phosphate-buffered saline, and then precipitated twice with 1ml 10% trichloroacetic acid. The precipitates were solubilized in 0.5 ml of 0.5% SDS/0.5M NaOH solution, and the extent of TdR incorporation was quantified using 75 µl of sample and 3 ml of scintillation fluid.

### Flow cytometric analysis

Cultured cells were washed and trypsinized. Cell suspensions were recovered and resuspended in the following fixing solution: 7ml 1X phosphate buffered saline, 2% bovine serum albumin, 5mM EDTA, 0.1% NaN_3_. 3ml of 100% ethanol was added drop wise. Fixed cells were centrifuged, washed, and then resuspended in 500µl sorting buffer: 1X phosphate buffered saline, 0.1% Triton-X 100, 2% bovine serum albumin, 5mM EDTA, 40µg/ml propidium iodide, 100µg/ml RNAse A, and incubated at 37C for 30 min. The cells were filtered through 70-µm mesh to remove cell aggregates. The DNA content was analyzed by flow cytometry (FACSCalibur; Becton Dickinson), and percentages of cells within each phase of the cell cycle were determined using WinCycle software (Phoenix Flow Systems).

## Results

### Growth factor and amino acids deprivation, as well as mTOR inhibition induce G1 cell cycle arrest

**Figure 1:**
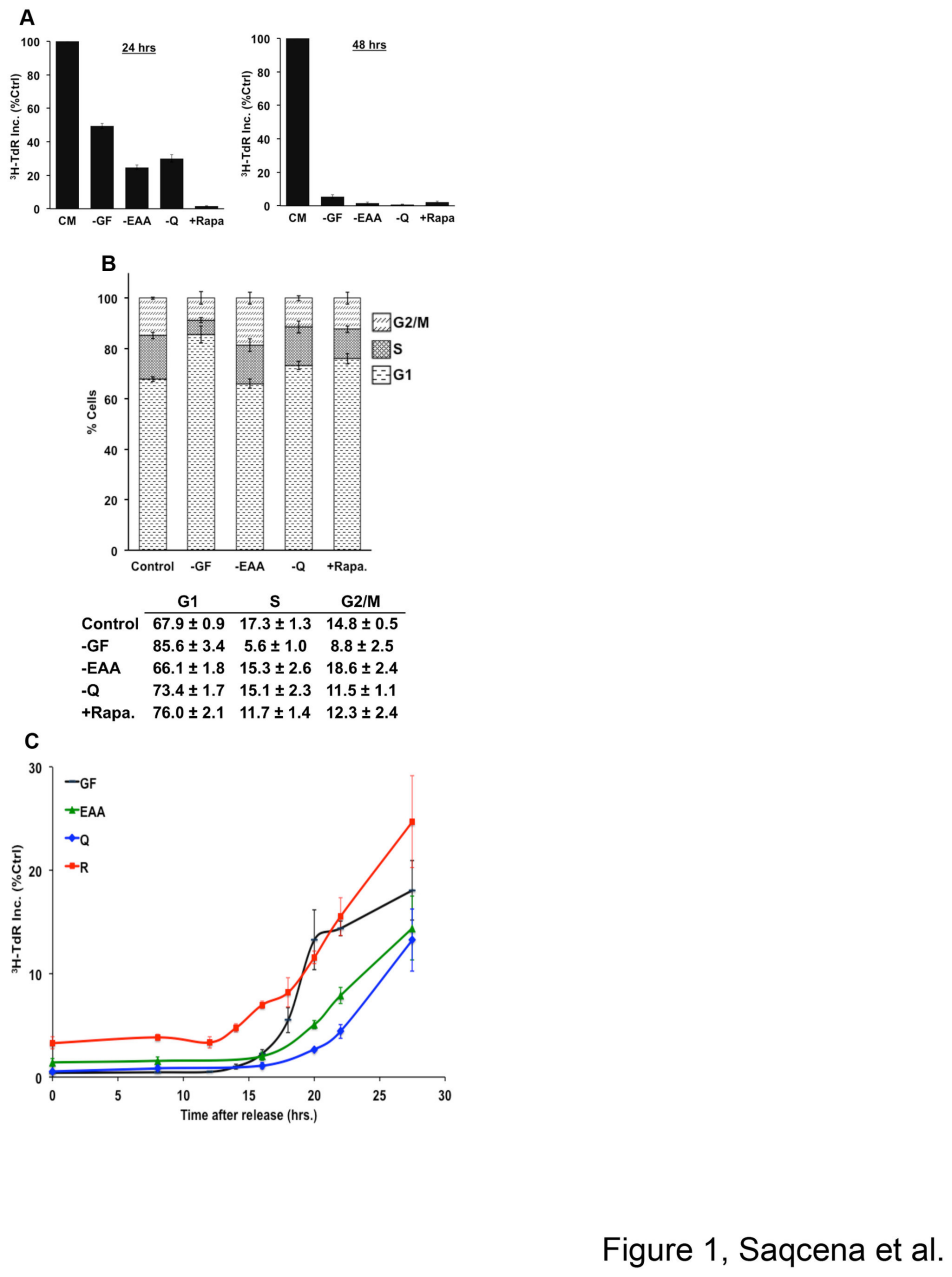
Growth factor and amino acid deprivation, as well as mTOR inhibition induce G1 cell cycle arrest. (**A**) BJ hTERT cells were plated at 20% confluence in DMEM containing 10% FBS for 24 hr at which time they were shifted to complete medium (CM) or various blocking conditions [-GF, -EAA, -Q, +Rapamycin (20 µM)] for 24 or 48 hr. The blocking conditions for Q used DMEM lacking Q; and for EAA, DMEM lacking Leu, Lys, and Arg as described in Material and Methods. The CM contained 10% dialyzed FBS (DFBS) instead of 10% FBS. Cells were labeled with [^3^H]-TdR for the final 24 hr of treatment, after which the cells were collected and the incorporated label was determined by scintillation counting as described in Materials and Methods. Values were normalized to the cpm for CM, which was given a value of 100%. Total cpm for the CM controls was 60,512 +/- 6529 for the 24 hr time point and 80,427 +/- 3567 for the 48 hr time point. Error bars represent the standard deviation for experiments repeated at least two times. (**B**) BJ cells were plated and shifted to CM or various blocking conditions for 48 hr as in (**A**), after which the cells were harvested and analyzed for cell cycle distribution by measuring DNA content/cell as described in Materials and Methods. Error bars represent the standard error from independent experiments repeated four times. (**C**) To investigate the kinetics for progression into S-phase, BJ cells were plated and shifted to blocking conditions for 48 hr as in (**A**). Cells were subsequently released by shifting to complete medium, and pulsed with [^3^H]-TdR at the indicated time points for 1 hr – after which the cells were collected and the incorporated label was determined. Error bars represent the standard error of mean for experiments repeated three times.

To characterize the G1 arrest induced by GF and nutrient deprivation, and mTOR inhibition, we used the human foreskin fibroblast BJ hTERT cell line, which has been immortalized by introduction of telomerase to prevent replicative senescence [10]. BJ hTERT cells were shifted to medium lacking GF, EAA, Q, or complete medium containing 20 µM rapamycin for 24 or 48 hr. We demonstrated previously that complete G1 arrest in response to rapamycin required the high micro-molar doses that are able to suppress phosphorylation of the mTORC1 substrate eukaryotic initiation factor 4E-binding protein-1 (4E-BP1) [11]. In that study we had shown that 20 µM rapamycin is required to completely suppress TdR incorporation in MDA-MB-231 and MCF7 breast cancer cells. Rapamycin dose response similar to MCF7 cells was obtained with BJ cells (data not shown). We also demonstrated that the effect of rapamycin was specific for mTORC1 and not due to off-target effects. To monitor progression into S-phase, the cells were labeled using [^3^H]-TdR for the final 24 hr of treatment. As shown in Figure 1A, GF, EAA, or Q deprivation causes 50-70% decrease in [^3^H]-TdR incorporation in initial 24 hr. However, by 48 hr, TdR incorporation reduced to less than 5% of control, indicating complete cell cycle arrest under these conditions. Rapamycin caused complete arrest at both 24 and 48 hr of treatment.

We next examined cell cycle distribution in BJ cells by measuring DNA content using flow cytometry. The cells were placed in various blocking conditions for 48 hr, fixed and stained using propidium iodide and analyzed by FACS. The cells had a marked increase in G1 cell population at the expense of S- and G2/M-phase cells upon serum deprivation and to a lesser extent with rapamycin (Figure 1B). However upon EAA and Q deprivation, BJ cells maintained their S-phase DNA content despite the observation in Figure 1A that there was no DNA synthesis after 48 hr of deprivation. This would indicate that for cells in S-phase, the lack of either EAA or Q prevents cells from progressing out of S-phase (Figure 1B). While rapamycin led to an increase in the percentage of G1-phase cells and a decrease in the percentage of S-phase cells, the percentage of G2/M-phase cells remained constant – indicating that rapamycin may also retard progression through G2 and or M.

To determine whether cells are capable of re-entry into cell cycle, we measured the kinetics for progression into S-phase upon release from various arrested states. In brief, cells were placed in various blocking conditions for 48 hr. Cells were then released from the block by replacing with complete medium, and pulsed with [^3^H]-TdR for 1 hr at indicated time points (0 to 28 hr). As shown in Figure 1C, cells starting from G0 (GF deprivation) took approximately 16 hr to enter the S-phase. Cells starting from rapamycin block also entered the S-phase after 16 hr. Surprisingly, cells starting from EAA or Q block began synthesizing DNA with a longer lag phase of 18 to 20 hr. While there are a small fraction of cells in S-phase (5-15%) with various blocking conditions as seen by flow cytometry (Figure 1B), the prolonged lag phase along with low baseline and sharp transition indicates that TdR incorporation occurs predominantly from G1-phase cells released from blocking conditions into S-phase (Figure 1C). Our observation for the time required to traverse from G0 to S-phase is similar to what has been described previously [12–14]. Thus, the kinetic analysis shows that cells are able re-enter the cell cycle upon release from various blocking conditions. These data also reveal differences in recovery times after being subjected to EAA, Q and GF deprivation; however they do not provide insight as to the temporal relationships of the different blocking mechanisms.

### GF, EAA, Q, and rapamycin mediated G1 cell cycle arrests are distinct and distinguishable

**Figure 2:**
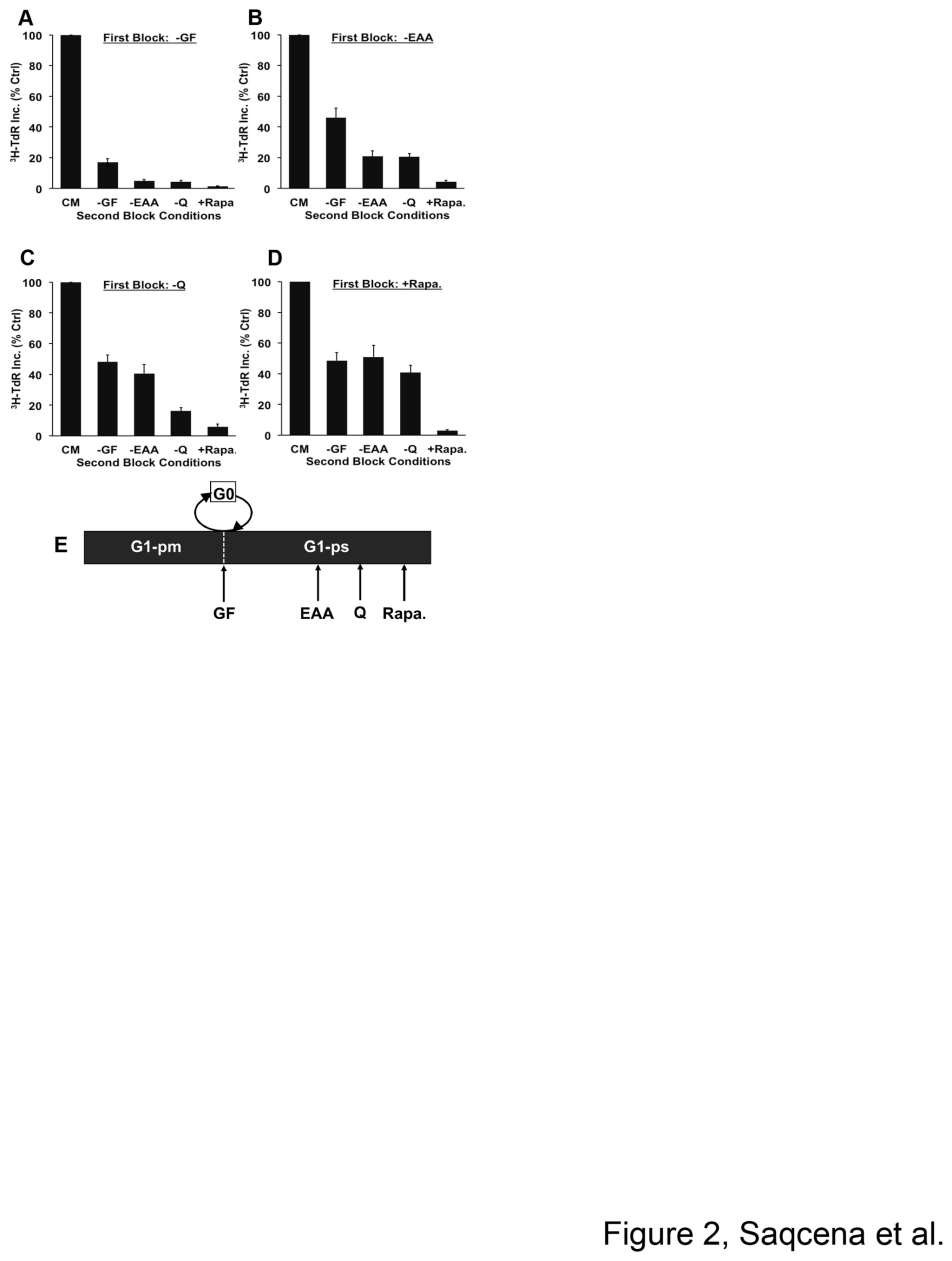
GF, EAA, Q, and rapamycin mediated G1 cell cycle arrests are distinct and distinguishable. (**A**–**D**) BJ hTERT cells were plated and shifted to various first blocking conditions for 48 hr as in Figure 1A. The cells were subsequently shifted to CM or different second block conditions containing [^3^H]-TdR for 24 hr, after which the cells were collected and the incorporated label was determined. Error bars represent the standard error for the experiment repeated at least four times. (**E**) Schematic model showing relative positions of different metabolic checkpoints relative to R (not drawn to represent precise time scales). G1-pm is post-mitotic phase in G1, G1-ps is pre-S phase of G1 [4].

In order to distinguish G1 cell cycle arrest caused by different blocking conditions, we performed a series of sequential blocking experiments. In brief, cells were exposed to various blocking conditions for 48 hr to cause complete arrest. At this point, the first block was removed and a second block was applied along with [^3^H]-TdR for 24 hr. If the second block applied is either at the same point or downstream of the first block, then [^3^H]-TdR incorporation should not occur. However, if the second block site is upstream of the first block, then the cells should progress into S-phase and incorporate the label. The extent of [^3^H]-TdR incorporated by cells released into complete medium after various first blocks was considered to be 100%. As shown in Figure 2A, when GF deprivation was applied as the first block and when either EAA or Q deprivation, or rapamycin treatment were applied as second blocks, there was very little [^3^H]-TdR incorporation- indicating that the GF arrest site is either upstream or at the same site as other blocking conditions. When EAA deprivation was applied as the first block (Figure 2B), followed by a second block of GF deprivation, increased [^3^H]-TdR incorporation was seen. However, with either Q deprivation or rapamycin treatment as the second block, no significant [^3^H]-TdR incorporation was observed. When Q deprivation was applied as the first block (Figure 2C), only rapamycin treatment as the second block prevented progression into the S-phase, whereas a second block of GF or EAA deprivation failed to arrest the cells. Lastly, when rapamycin treatment was applied as a first block followed by GF, EAA, or Q deprivation as the second block, there was an increase in [^3^H]-TdR incorporation in all the cases, indicating that all the blocks are upstream of rapamycin arrest site (Figure 2D). Taken together, the data indicates that the arrest sites mediated by various blocking conditions are distinguishable, in the order of GF → EAA → Q → mTOR (Figure 2E).

### Temporal mapping of the G1 cell cycle checkpoints

**Figure 3:**
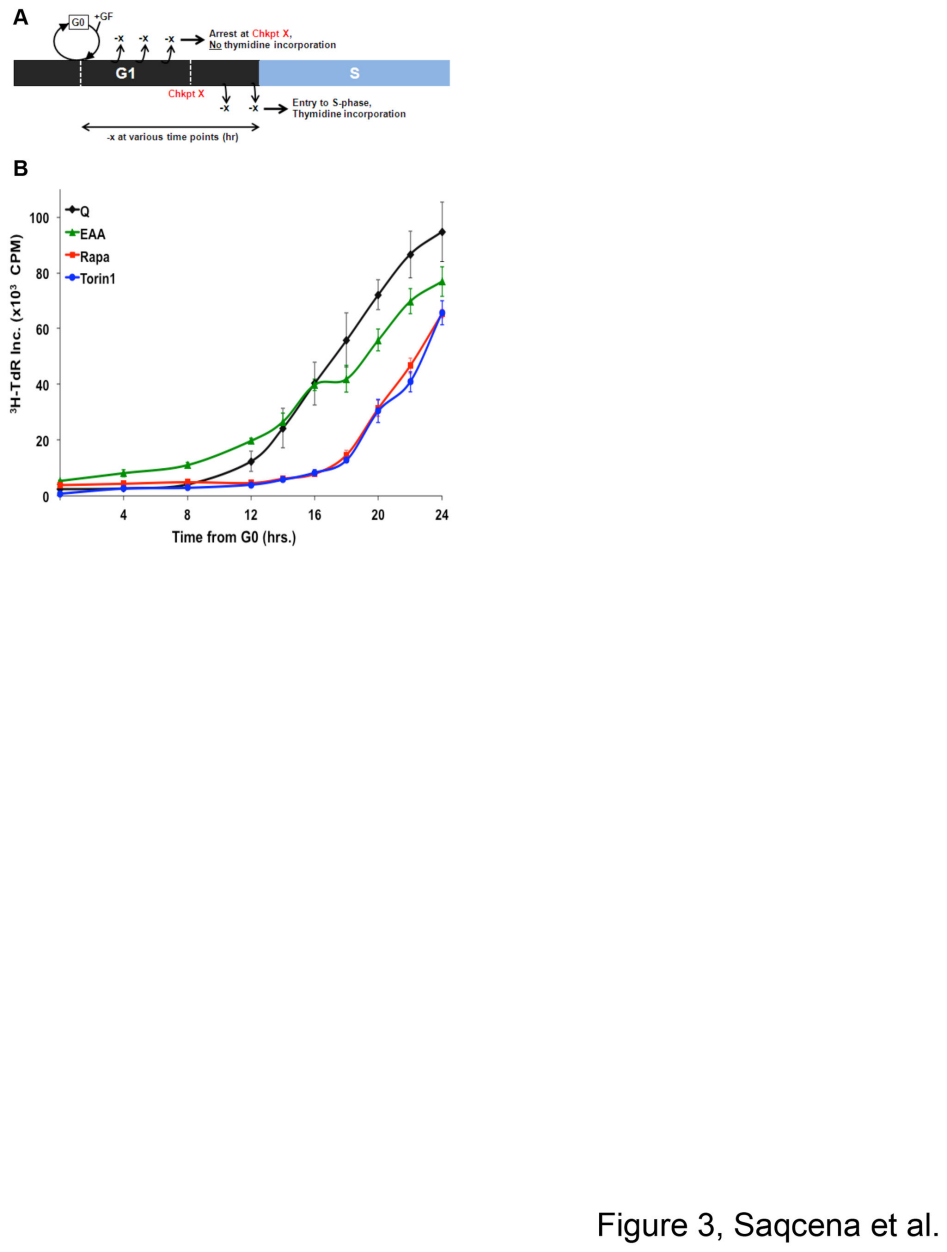
Temporal mapping of the metabolic checkpoints from G0. (**A**) Schematic representation of the experiment shown in (**B**). (**B**) BJ cells were plated as in Figure 1A for 24 hr. Cells were synchronized in G0 by shifting to DMEM+1mM Q lacking GF for 48 hr. The cells were released from G0 by shifting to CM containing DMEM (1mM Q) and 1 µCi/ml [^3^H]-TdR. Various blocking conditions along with [^3^H]-TdR were applied at indicated time points. After 36 hr from the release from G0, cells were collected and the incorporated label was determined. This experiment utilized DMEM with reduced Q (1 mM vs. 4 mM) because Q withdrawal following DMEM with high Q did not give strong G1 arrest. Error bars represent the standard error for experiments repeated three times.

To better understand the temporal map shown in Figure 2E, we examined the ability of EAA, Q, and rapamycin to block G1 cell cycle progression after release from G0. Cells were synchronized in G0 using serum deprivation for 48 hr. The cells were reinitiated into cell cycle by providing complete medium and [^3^H]-TdR. At indicated time points, cells were shifted to various blocking conditions to determine the point when blocking no longer prevented entry into S-phase (schematic shown in Figure 3A). As shown in Figure 3B, starting from G0, EAA or Q withdrawal until 12 hr caused the cells to arrest in G1, after which their withdrawal did not arrest the cells as evidenced by increased TdR incorporation. Addition of rapamycin continued to suppress TdR incorporation until 16 hr after release from G0, as did a catalytic inhibitor of mTOR – Torin1. This suggests that EAA and Q checkpoints are 12 hr from G0 and are upstream from rapamycin-mediated arrest, which apparently is very close to the G1/S border since it takes 16 hr from the time of restoring GF to increased TdR incorporation (Figure 1C).

### Restriction point and metabolic checkpoint arrest lead to differential patterns of cell cycle regulator expression and phosphorylation

**Figure 4:**
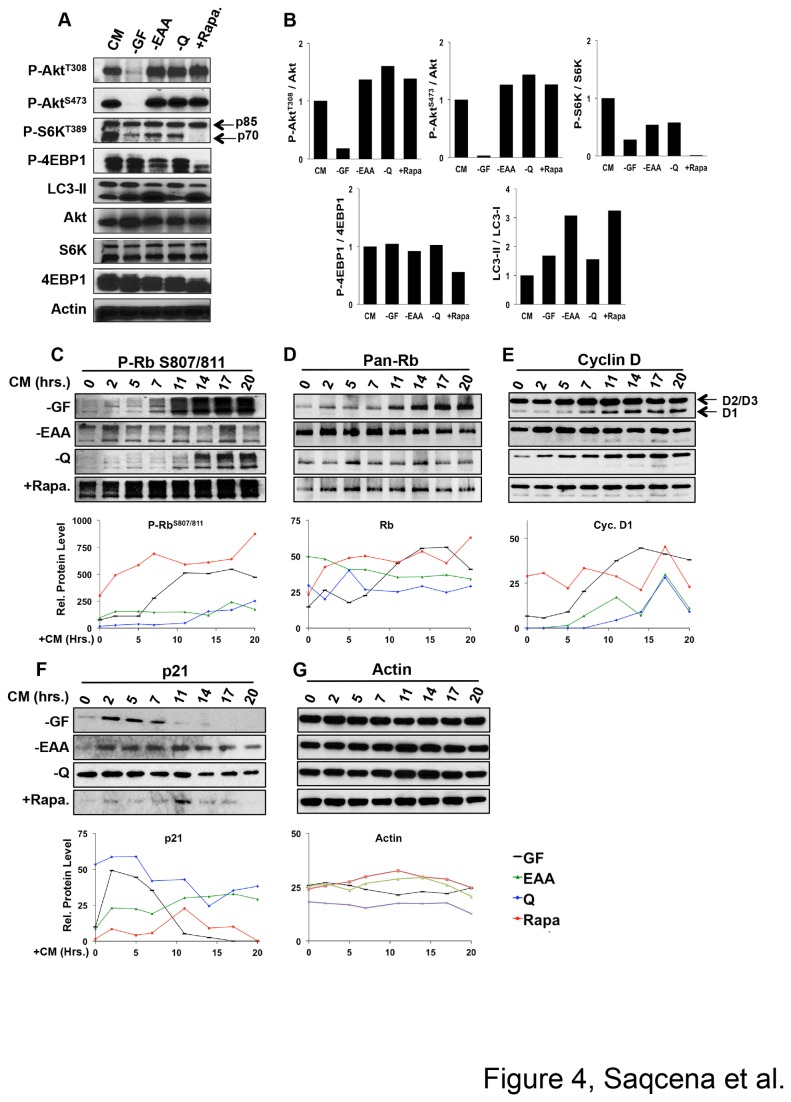
Restriction point and metabolic checkpoint arrest lead to differential patterns of cell cycle regulator expression and phosphorylation. (**A**) Cells were plated at 30% confluence in 10-cm plates in DMEM containing 10% FBS. After 24 hr, the cells were shifted to CM or blocking conditions for 4 hr, at which time the cells were harvested and the levels of the indicated protein or phosphoprotein was determined by Western blot analysis. The data shown are representative of experiments repeated at least two times. (**B**) Quantitative analysis of relative protein levels for Western blots shown in (**A**) using ImageJ software. (**C**–**F**) BJ cells were plated and shifted to various blocking conditions for 48 hr as in Figure 1A. The cells were subsequently released by shifting to CM, and the cells were harvested and lysates collected at indicated time points. The levels of the indicated protein or phosphoprotein were determined by Western blot analysis. The data shown are representative of experiments repeated at least two times. Also shown in the line graphs are the kinetic analyses of relative protein levels normalized to actin and quantitated using ImageJ.

The data in Figures 2 and 3 indicate a temporal difference in the ability of GF, EAA, and Q deprivation, and rapamycin to arrest cells in G1. To further establish that the cell cycle checkpoints are distinct – especially between the EAA and Q checkpoints, which apparently are temporally very close to each other – we examined the impact on cell cycle regulatory signals. A key cell cycle regulatory signaling pathway is mediated by phosphatidylinositol-3-kinase (PI3K) and Akt signals that impact on mTOR [15]. Akt is phosphorylated at Thr308 in response to GF stimulation of PI3K activation [16]. As expected, GF deprivation led to a decrease in Akt phosphorylation at Thr308 (Figure 4A and 4B). There was also a decrease in Akt phosphorylation at Ser473 - a downstream target of mTORC2 [17]. There was a marked decrease in phosphorylation of p70-S6 kinase (p70^S6K^) and a smaller decrease in the phosphorylation of4EBP1 upon GF deprivation. In contrast, EAA and Q deprivation had no effect on Akt phosphorylation at either Thr308 or Ser473, indicating no effect on PI3K or mTORC2 activity (Figure 4A and 4B). However, both EAA and Q deprivation did suppressp70^S6K^ phosphorylation. Interestingly, EAA deprivation suppressed 4E-BP1 phosphorylation, whereas Q deprivation did not –revealing a differential impact on mTORC1 in response to EAA and Q deprivation (Figure 4A and 4B). Treatment of cells with rapamycin led to no noticeable change in Akt phosphorylation at either Thr308 or Ser473. As expected, rapamycin suppressed the phosphorylation of both mTORC1 substratesp70^S6K^ and 4EBP1. We also examined the impact of nutrient and GF deprivation on autophagy by looking at increased levels of the autophagy marker LC3-II. Significantly, EAA, but not Q deprivation increased LC3-II levels. As expected, rapamycin treatment, which is known to induce autophagy [18], also led to increased levels of LC3-II (Figure 4A and 4B). Thus, although all of the conditions used here cause G1 cell cycle arrest, they impact differentially on PI3K and mTOR kinase activity supporting the hypothesis that the checkpoints identified represent distinct sites in G1 – especially between the two amino acid sites.

We next examined the impact of different blocking conditions on known G1 cell cycle regulators. For this approach, cells were placed under various blocking conditions for 48 hr and subsequently released by restoring complete medium. Cell lysates were collected at indicated time points and analyzed for phosphorylated-Rb (P–Rb), Rb, cyclin D, and p21 using Western blot analysis. For cells arrested by GF deprivation, we see much less Rb protein and P–Rb Ser807/811 at time 0, but there was a dramatic increase in both Rb protein and P–Rb Ser807/811 levels from 11hr onwards (Figure 4C and 4D). A similar Rb profile was seen with cells starting from a Q deprived state. However, for cells starting from the EAA deprived state, there were high levels of Rb at time 0 that did not change much after restoring the EAA. This effect was clearly distinct from that seen with Q deprivation, where there were much lower levels of both Rb and P–Rb. Rapamycin treatment did not significantly reduce the levels of Rb protein or P–Rb - indicating that cells had arrested in late-G1 where Rb is already hyperphosphorylated. Passage through R correlates with an increase in cyclin D levels [19]. Upon restoration of complete medium to GF-deprived cells, there was a significant increase in the level of cyclin D1 between 5 and 14 hr (Figure 4E). In contrast, cells starting from EAA, Q, or rapamycin blocking conditions showed lesser changes in cyclin D1 levels – indicating a clear distinction between the GF-dependent R and the later nutrient-dependent metabolic checkpoints. Cells starting from all blocking conditions showed very similar cyclin E profiles, with cyclin E levels increasing from 11 hr onwards (data not shown). The CDK inhibitor p21 plays complex roles in controlling G1 cell cycle progression [20,21]. For cells starting from GF-deprived state, there was very little p21 at time 0 but its level increased significantly by 2 hr and then dropped after 7hr (Figure 4F). The drop in p21 levels coincided with the increase in cyclin D levels and Rb phosphorylation at S807/811 (Figure 4C and 4E). With EAA and Q deprivation, there were very low levels of p21 at time 0 for EAA and high levels with Q – again clearly distinguishing these two checkpoints. With rapamycin block there was very little p21 at time 0 that was maintained over the 20 hr time course. Collectively, the data in Figure 4 reveal differential impact of various blocking conditions on the expression and phosphorylation of cell cycle regulatory proteins. While the data do not provide mechanistic insight into cell cycle arrests mediated by different blocking conditions, they clearly establish that the cell cycle arrest caused by various blocking conditions represent unique cell cycle checkpoints.

### Metabolic checkpoints are dysregulated in cancer cells

**Figure 5:**
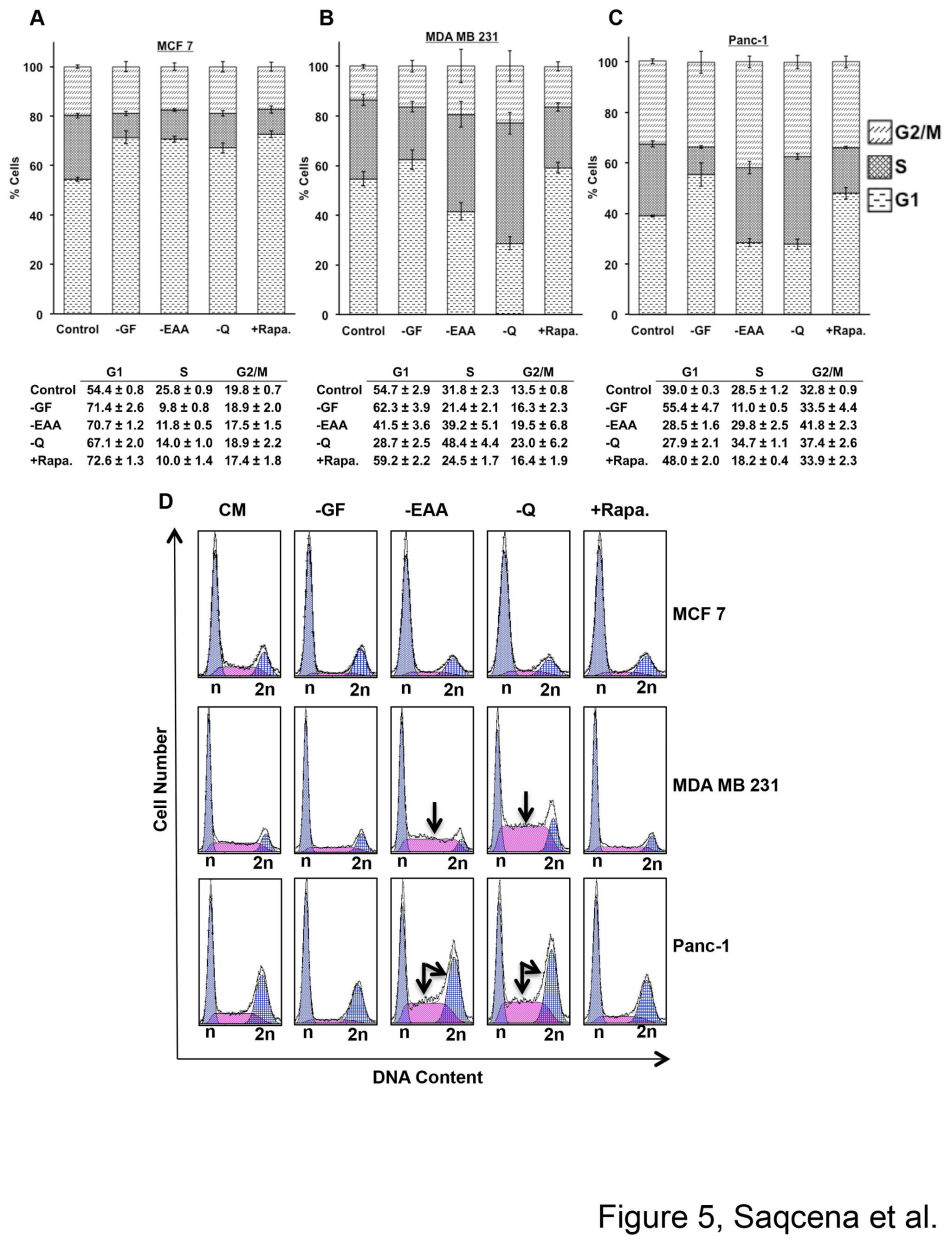
Metabolic checkpoints are dysregulated in cancer cells. MCF7 (**A**), MDA-MB-231 (**B**), and Panc-1 (**C**) cells were plated at 20% confluence in 10-cm plates in DMEM containing 10% FBS. After 24 hr, the cells were shifted to CM or various blocking conditions for 48 hr, at which time the cells were harvested, fixed, stained with propidium iodide, and analyzed for distribution in different phases of cell cycle by measuring DNA content/cell as described in Materials and Methods. Error bars represent the standard error from independent experiments repeated four times. Table with the mean and standard error for the graphs is also shown. (**D**) Representative flow histograms showing increases in S- and G2/M-phase cell population upon EAA and Q deprivation in MDA-MB-231 and Panc-1 cells.

Complementing mutations required for transformation [2] suggest that in addition to GF autonomy, cancer cells may also have dysregulated metabolic checkpoints to allow progression through both R and the late-G1 metabolic cell growth checkpoints. To further characterize the impact of GF and nutritional inputs in cancer cells, we examined cell cycle distribution in three human cancer cell lines. Cells were placed in various blocking conditions for 48 hr and analyzed by flow cytometry. MCF7breast cancer cells had a marked increase in G1 cell population at the expense of S-phase cells with serum or amino acid deprivation and rapamycin treatment (Figure 5A and 5D top panel). The G2/M-phase cells remained constant indicating a G2/M-phase arrest of cells as was observed for BJ cells deprived of EAAs in Figure 1B. The BJ cells deprived of Q displayed an S-phase and G1 phase arrest, but not a G2/M arrest (Figure 1B) indicating a differential sensitivity to EAA and Q for the MCF7 breast cancer cells. In stark contrast to both the BJ cells and the MCF7 cells, MDA-MB-231 breast and Panc-1 pancreatic cancer cell lines displayed a dramatic loss of any G1-phase arrest in response to both EAA and Q deprivation(Figure 5B, 5C, and 5D middle and lower panel). Both of these cell lines retained a G1 arrest in response to serum withdrawal. This observation supports the hypothesis that the cells have a mechanism for arresting in S- and G2/M-phase upon EAA and Q deprivation. Importantly, it also demonstrates that the ability of EAA and Q to arrest in G1 has been lost in the MDA-MB-231 and Panc-1 cells. Rapamycin caused an increase in the G1 cell population in all the cell lines tested, indicating that inhibition of mTORC1 activity is sufficient to cause G1 cell cycle arrest. As with the BJ cells (Figure 1B), rapamycin also appeared to retard cell cycle progression in G2/M. These observations indicate that EAA and Q sensing acts through separate mechanism than rapamycin treatment or GF sensing to cause G1 arrest, and that metabolic deregulation in the MDA-MB-231 and Panc1, but not the MCF7, cells causes override of G1 cell cycle arrest upon amino acid deprivation.

## Discussion

In this report, we have provided evidence that the GF-mediated R and nutrient-mediated metabolic checkpoints are distinct and distinguishable. Sequential blocking experiments show that R is upstream of two amino acid checkpoints that are upstream from a checkpoint mediated by mTOR. Although the checkpoints mediated by EAA and Q were temporally close, they could be distinguished by sequential blocking and by distinct profiles of cell cycle regulatory protein expression and phosphorylation. Suppression of mTOR with rapamycin blocked cell cycle progression significantly later in G1 than amino acid deprivation. Collectively, this study distinguishes the GF-dependent R, which assesses whether it is appropriate for the cell to divide, from a series of metabolic checkpoints late in G1 that determine whether division is feasible. In addition to mediating unique late-G1 checkpoints, our data also reveals novel sensing requirements for EAA and Q in S- and G2/M-phase of the cell cycle. Using Swiss 3T3 cells, Yen and Pardee had previously found that GF deprivation led to mid-G1 arrest whereas isoleucine deprivation caused late-G1 and S-phase arrest [22]. This result more closely approximates the findings reported here with the human BJ fibroblasts.

It was somewhat surprising that inhibiting mTOR blocked cell cycle progression downstream of EAA. It is well established that mTORC1 is responsive to EAA [23]. Thus, it was anticipated that the absence of EAA would block cell cycle progression at the same place as rapamycin. This was clearly not the case – there was a two-hour difference in the time it took for EAA deprivation to no longer prevent progression to S-phase relative to rapamycin (Figure 3B). However, mTORC1 is also responsive to glucose [24], ATP levels [25], and phosphatidic acid [26], a critical intermediate in the synthesis of membrane lipids [27–29]. Thus, mTOR may not be fully active until it has sensed sufficient glucose, ATP, lipids, in addition to EAA. Thus mTOR likely serves as a master regulator that senses complete nutritional sufficiency before committing to replicating the genome.

An important conclusion from this study is the distinguishing of growth factor-dependent R in mid-G1 from late-G1 metabolic checkpoints that control entry into S-phase. The point in G1 where the cells are no longer sensitive to the withdrawal of growth factors (R) was mapped by Zetterburg and colleagues to about 3.5 hr in virtually all mammalian cells tested [4]. The metabolic checkpoints downstream from R in this report are similar to a series of checkpoints in yeast collectively known as START [7,30], where nutritional sufficiency is evaluated in a TOR-dependent manner in yeast [8,9]. R has commonly been referred to as the mammalian equivalent of START, but as shown here, the metabolic checkpoints that correspond with START are clearly distinguishable from the growth factor-dependent R. It is likely that R evolved much later than START as a means for multicellular organisms to regulate proliferation through intercellular communication.

Part of the controversy over the location of a growth factor-dependent R is that different groups have reported responsiveness to growth factors later in G1 than described by Zetterberg [4]. Notably Pledger and Stiles reported that PDGF could stimulate quiescent cells to “competence” with a short duration of treatment [31]. These competent cells could then be induced to progress through the remainder of G1 by “progression” factors like insulin-like growth factor-1 (IGF1) [31]. Similar studies with hepatocytes induced from quiescence showed a growth factor dependence that likely was extended into later stages of G1 [32,33]. The major distinction between these studies and Zetterberg’s work was that the Zetterberg study followed cells from mitosis, whereas the other studies looked at cells leaving quiescence. Thus, cells starting from quiescence or G0 and cells starting from mitosis apparently have different needs for progression to S-phase. What was clear from the Zetterberg study was that after approximately 3.5 hr post mitosis, if serum growth factors were removed, cells did not enter quiescence and proceeded through S-phase to mitosis without any additional growth factor stimulation. In our double block experiments (Figure 2), the cells arrested by amino acid depletion or rapamycin could proceed to S-phase in the absence of growth factors upon restoration of amino acids or removal rapamycin. This is especially relevant for the mTOR checkpoint, since mTOR is activated in response to IGF1 and other growth factors. Importantly, the cells that arrested in G1 in response to amino acid deprivation and rapamycin, like in the Zetterberg study, were coming from mitosis, not quiescence, and therefore did not need growth factors to proceed to S-phase. This would indicate that under conditions where cells have passed through mitosis and avoided quiescence, mTOR does not need additional growth factor stimulation. However, this is apparently not the case when cells are coming out of quiescence where further stimulation of mTOR by IGF1 may be required.

GF autonomy is one of the more significant hallmarks in cancer [34]. However, it has been suggested that mutations leading to elevated mTOR kinase activity are the most common mutations in observed human cancer [35,36]. Moreover, dysregulation of cellular metabolism is considered as an emerging hallmark of cancer [37]. Several oncogenes and survival signals have been shown to directly upregulate glycolytic enzymes and induce metabolic reprogramming [38–40]. Consistent with this emerging role for metabolism in cancer cells, we have demonstrated here that nutrient sensing metabolic checkpoints are dysregulated in cancer cells. Surprisingly, MDA-MB-231 breast cancer cells and Panc-1 pancreatic cancer cells deprived of EAA and Q arrested in S and/or G2/M-phase – indicating an override of the G1 arrest observed in normal BJ fibroblasts and MCF7 breast cancer cells. Significantly, both the MDA-MB-231 and Panc1 have mutant K-Ras. Thus, the late-G1 metabolic checkpoints, like R, are apparently dysregulated as well – perhaps as a consequence of mutant K-Ras signals. Of interest was the apparent “freeze” in cell cycle progression in the BJ cells in response to amino acid deprivation – indicating that collection of cells in S- and G2/M-phase in cancer cells is a property of normal cells. In addition to genetic defects that confer autonomy to GF signaling, we hypothesize that specific genetic mutations override the late-G1 nutritional checkpoints causing them to arrest in S- and G2/M-phase of the cell cycle, where we have shown that additional amino acid sensing occurs through as yet unknown mechanisms. Cancer cells arrested in S- and G2/M-phase are uniquely sensitive to the apoptotic insult of DNA damaging agents. Thus, synthetic lethality created by interfering with Q utilization and phase-specific cytotoxic drugs could provide novel therapeutic opportunities that kill cells arrested in S- and G2/M-phase.
